# Biological activity comparison between ciprofloxacin loaded to silica nanoparticles and silver nanoparticles for the inhibition of *Brucella melitensis*

**DOI:** 10.14202/vetworld.2024.407-412

**Published:** 2024-02-20

**Authors:** Saif Aldeen Jaber, Mohamed J. Saadh

**Affiliations:** 1 Department of Pharmacy, Faculty of Pharmacy, Middle East University, Amman, Jordan; 2 Applied Science Research Centre, Applied Science Private University, Amman, Jordan

**Keywords:** antimicrobial activity, brucellosis, minimum inhibitory concentration, nanoparticles

## Abstract

**Background and Aim:**

*Brucella melitensis* is responsible for brucellosis, a highly contagious, life-threatening disease that has a high impact in low- and middle-income countries. This study aimed to compare silica nanoparticles (SiO-NPs) loaded with ciprofloxacin with silver nanoparticles (AgNPs) loaded with ciprofloxacin to evaluate the possible replacement of silver by silica to enhance biological activity and reduce cytotoxicity.

**Materials and Methods:**

SiO-NPs and AgNPs loaded with ciprofloxacin were characterized using ultraviolet spectroscopy, scanning electron microscopy, and dynamic light scattering microscopy for size demonstration and loading efficiency. Both nanoparticles were treated with *B. melitensis* Rev 1 to evaluate their biological activity. Nanoparticle toxicity was also evaluated using cytotoxicity and hemolysis assays.

**Results:**

SiO-NP was found to have a smaller size (80 nm) and higher loading efficiency with polydispersity index and zeta potential of 0.43 and 30.7 mV, respectively, compared to Ag-NP (180 nm and 0.62 and 28.3 mV, respectively). SiO-NP was potent with a minimum inhibitory concentration of 0.043 μg/mL compared to Ag-NP (0.049 μg/mL), with a lower cytotoxicity and hemolysis activity.

**Conclusion:**

SiO-NP, as a drug delivery system for ciprofloxacin, has better antimicrobial activity against *B. melitensis* with lower cytotoxicity and hemolysis activity. These results can be attributed to the enhanced physical characterization and better loading efficiency when compared to Ag-NP.

## Introduction

Microbial resistance has recently emerged due to patients’ uncontrolled use of antibiotics, especially in middle- and low-income countries [1]. Bacterial infections caused by pathogenic bacteria are responsible for millions of annual deaths worldwide in addition to billions of dollars lost from the health sector [2]. Multidrug-resistant bacteria have pushed pharmaceutical companies and research laboratories to find new antimicrobial compounds or new drug delivery methods to increase drug targeting or potency [3]. Nanomedicine techniques have been found to enhance drug targeting and potency, especially for antimicrobial drugs that show great antimicrobial activity against severe microbial infection [4]. Many materials, such as silica and silver nanoparticles (AgNPs), have already been used to produce nanoparticles used in drug delivery systems. AgNPs are metallic nanoparticles that have previously been studied due to their optical, electronic, and magnetic characteristics [5]. Different research laboratories have prepared many nano-systems to enhance either the biological activity of the drug or/and to enhance drug selectivity to reduce its side effects [6]. Carbon nanotubes are nanoparticle systems with diameters of 0.5–3 nm and lengths of 100 nm that can form single or multilayer tubes [7]. Carbon nanotubes enhance the cytoplasmic and nucleus penetration for gene delivery [7]. A dendrimer is another type of nanomedicine delivery method that consists of a core, branch, and surface with a size below 10 nm [8]. Dendrimers are used by different pharmaceutical research laboratories to enhance drug targeting and control drug release [8]. Liposomes are the third type of nano-delivery system made of phospholipids with a size between 50 and 100 nm [9]. Liposomes are generally used for gene delivery and long circulation [9]. Polymeric nanoparticles are nanoparticles made of a biodegradable polymer with a size of 100 nm or smaller [10]. Polymeric nanoparticles are actively or passively used for the drug delivery of bioactive compounds (natural, semi-synthetic, and synthetic) [10]. Finally, metallic nanoparticles are very stable nano-systems made of metallic materials that are used for various drug and gene delivery applications [11,12].

This type of nanoparticles has been used due to their effectiveness in the delivery of antibiotics to enhance their biological activity, especially due to their large surface area [13–15]. On the other hand, silica has been widely used in nanoparticle drug delivery systems owing to its large specific surface area, controllability of particle size, and biocompatibility with many drugs [16, 17]. The increase in antibiotic resistance has become a global problem because many microorganisms are resistant to different types of antibiotics. Therefore, alternative materials with high antibacterial activity, such as nanoparticles, have been explored. AgNPs and silica nanoparticles (SiO-NPs) have attracted significant attention due to their unique properties and ability to inhibit microbial growth [13, 14, 18].

In this study, multiple comparisons have been performed between AgNPs and SiO-NPs loaded with ciprofloxacin using optical absorption, light scattering, scanning electron microscopy (SEM), and different biological assays to determine their biological activity against *Brucella melitensis*. This study aimed to confirm the physical characteristics and biological activities of SiO-NPs and AgNPs and show the higher safety of using SiO-NPs over AgNPs.

## Materials and Methods

### Ethical approval

Ethical committee approval was obtained from the Ethical Committee of Middle East University, Amman, Jordan (approval no. 2021.07).

### Materials

*B. melitensis* Rev 1 was obtained from JOVAC Bio Industry Center, Amman, Jordan. Ciprofloxacin (99.9 purity) was obtained from MS Pharmaceutical Company. Silver nitrate (AgNO_3_), sodium borate (NaBH_4_), tetraethyl orthosilicate (TEOS) (99.9%), absolute ethanol (99.5%), and ammonium hydroxide (NH_3_ 25%) were purchased from Sigma-Aldrich, USA. Human kidney 2 (HK-2) and prostate (PN2TA) cell lines were obtained from Cell Bank and Stem Cell Bank, Shanghai Institute for Biological Sciences, Chinese Academy of Sciences, CHEN Yuelei.

### Study period and location

The study was conducted from April 2023 to June 2023 at Middle East University, Amman, Jordan.

### Preparation of AgNPs

A 5 mL aliquot of 0.1 mM ciprofloxacin HCl solution in phosphate buffer (pH 7.4) was mixed for 10 min with 5 mL aliquot of 0.1 mM AgNO_3_ solution, followed by the addition of 20 μL of freshly prepared NaBH_4_ to the mixture. The mixture was centrifuged at 12,000× *g* for 1 h. The supernatant was collected to calculate the amount of unloaded drug.

### Preparation of SiO-NPs

**Figure-1:**
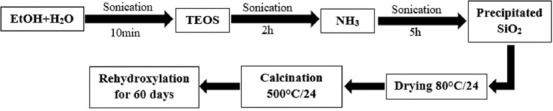
Schematic diagram showing the preparation of silica nanoparticles.

SiO-NPs were prepared using Stöber process loaded with ciprofloxacin, as shown in Figure-1 [19]. After the addition of NH_3_ to improve the activity of TEOS, 5 mL of 0.1 ciprofloxacin was added to the prepared SiO-NPs. The concentrations of other materials were added according to Table-1 with preparation parameters that presented the ideal concentration according to a previous study by Rahman *et al*. [19].

**Table-1:** Experimental parameters for silica nanoparticle preparations.

Parameters	Value
[TEOS] (mol/L)	0.80
[NH_3_] (mol/L)	1.87
*R* = ([H_2_O]/[TEOS])	37.0
Feed rate (mL/min)	0.03
Temperature (°C)	45 ± 5

TEOS=Tetraethyl orthosilicate

### Particle size, zeta potential, and polydispersity index (PDI)

The zeta potential and PDI were measured using dynamic light scattering (DLS, Zetasizer Nano NS, Malvern Instruments, Malvern, UK). In addition, particle sizes were calculated using SEM by dissolving the samples in purified water at a temperature of 250°C with an angle of 1730°. Similarly, the zeta potential was measured by laser Doppler anemometry at the same temperature with a purified water-adjusted conductivity (50 μS/cm) and 0.9% (m/V) NaCl solution using the same instrument. Each reported value represents the average of three measurements (mean ± standard deviation).

### Minimum inhibitory concentrations (MIC)

The MIC biological assay was performed for silica and AgNPs loaded with ciprofloxacin according to a previous method published by Saadh [15]. *B. melitensis* Rev 1 was incubated on *Brucella* broth for 18 h, followed by dilution to 10^6^ colony-forming units/mL using *Brucella* broth according to the recommendations of the Clinical and Laboratory Standards Institution. A concentration range of 0.01–100 μg/mL of ciprofloxacin, ciprofloxacin loaded on NP-AgNO_3_, and ciprofloxacin loaded on NP-SiO_3_ was added to 50 μL of diluted *B. melitensis* Rev 1, followed by incubation for 5 days at 37°C. The absorbance of each well was measured at 570 nm using a Thermo-lab Systems Reader (Finland). The positive control was 100 μL of *B. melitensis* broth, while the negative control was 50 μL of diluted *B. melitensis* Rev 1 and 50 μL of *Brucella* broth. Bacterial growth was examined using a direct observation method in the presence or absence of turbidity.

### Cytotoxicity assay

The cytotoxic effects of ciprofloxacin loaded on silica and AgNPs were evaluated using HK2-Vi and PN2TA cell lines. Cells were cultured in RPMI 1640 medium supplemented with 1% penicillin-streptomycin, 5% fetal bovine serum, and 1% L-glutamine. Cells were stored at 37°C in a cell culture incubator (gaseous composition: 95% air, 5% CO_2_). The AlamarBlue^®^ assay (Biosource International, Camarillo, CA, USA) was used to determine the cytotoxicity effect of ciprofloxacin loaded on silica and AgNPs. Ninety-six well plates were seeded with HK2-Vi or PN2TA cell line at a concentration of 2 × 10^4^ cells/mL for each well. Cells were cultivated 1 day before the addition of 1000, 500, 300, 50, 30, and 5 μg/mL of ciprofloxacin loaded on silica or AgNPs. Triton-X (4% v/v) was added as a positive control to the medium. Cells were incubated for 24 h, followed by the addition of 10% v/v AlamarBlue® solution into each well, followed by incubation for 6 h. The reduced form of resazurin is pink and extremely fluorescent, and the intensity of the fluorescence produced is proportional to the number of living cells that are respired. A wavelength of 570 nm was used for absorbance measurements.

### Erythrocyte hemolytic assay

An erythrocyte hemolytic assay was performed for both silica and AgNPs loaded with antibiotics to determine the possible hemolysis of human red blood cells. We performed a hemolytic test, as published by Hollmann *et al*. [20].

## Results

### Optical properties of nanoparticles

**Figure-2:**
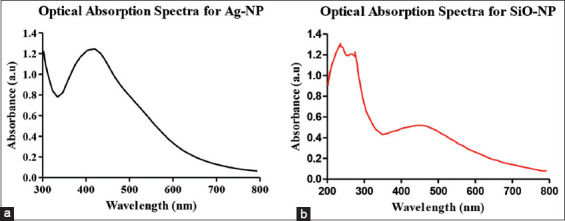
Optical absorption spectra for both silver nanoparticles (a) and silica nanoparticles (b) by surface plasmon resonance.

The optical absorbance of silver and SiO-NPs loaded with the drug showed different optical properties, as shown in Figure-2. AgNPs show a higher wavelength of approximately 420 nm, whereas nanoparticles formed from silica show a higher wavelength of approximately 250 nm.

### Particle size, PDI, and zeta potential

**Figure-3:**
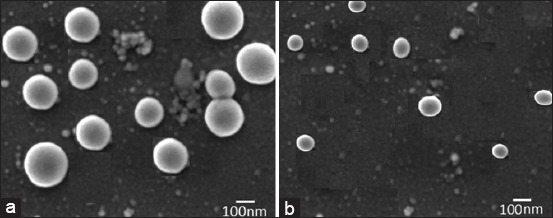
Scanning electron microscope results for ciprofloxacin loaded on both (a) silver and (b) silica nanoparticles.

As shown in Figure-3, SiO-NPs show particles with lower diameters with an average of 80 nm. On the other hand, when ciprofloxacin was loaded to AgNPs with an average diameter of 180 nm, a higher nanoparticle diameter was formed. The particle size distribution and SEM results are presented in Figure-3. In addition, DLS analysis revealed that both Ag-NP and SiO-NP had good PDI and zeta potential, as presented in Table-2.

**Table-2:** Physical characterization of formulated nanoparticles.

Formula	Main particle size (nm) ± SD	PDI ± SD	Zeta potential (mv) ± SD
SiO-NP	79.3 ± 3.3	0.43 ± 0.03	30.7 ± 1.4
Ag-NP	180.7 ± 6.1	0.62 ± 0.12	28.3 ± 2.8

Ag-NPs=Silver nanoparticles, SiO-NPs=Silica nanoparticles, PDI=Polydispersity index

### MIC

Table-3 presents the MIC values of ciprofloxacin and ciprofloxacin loaded on nanoparticles against *B. melitensis* Rev 1. AgNPs and SiO-NPs showed better biological activity against *B. melitensis* Rev 1 than free ciprofloxacin. Ciprofloxacin loaded on SiO-NPs showed higher biological activity (MIC = 0.043 μg/mL), whereas ciprofloxacin loaded on AgNPs showed lower biological activity (MIC = 0.049 μg/mL). On the other hand, ciprofloxacin alone has the lowest biological activity with a MIC of 0.73 μg/mL.

**Table-3:** MIC values of ciprofloxacin and ciprofloxacin loaded on nanoparticles against *Brucella melitensis* Rev 1.

Antibiotic	MIC ± Standard Deviation
Ciprofloxacin alone	0.73 ± 0.0091
Ciprofloxacin - Ag-NPs	0.049 ± 0.00023
Ciprofloxacin - SiO-NPs	0.043 ± 0.00029

MIC=Minimum inhibitory concentration, Ag-NPs=Silver nanoparticles, SiO-NPs=Silica nanoparticles

### Hemolytic activity

The hemolysis activity of ciprofloxacin loaded onto the nanoparticles is presented in Table-4. As shown in Table-4, both silver and SiO-NPs resulted in low hemolysis activity in erythrocytes. At a concentration of 100 μg/mL, ciprofloxacin loaded onto AgNPs showed higher activity (2% compared to 1% for SiO-NPs). On the other hand, at a higher concentration of 200 μg/mL, SiO-NPs loaded with ciprofloxacin exhibited a higher hemolysis activity of 12% compared to 9% produced by AgNPs loaded with ciprofloxacin.

**Table-4:** Hemolysis activity of ciprofloxacin loaded to nanoparticles.

Ciprofloxacin silver nanoparticles	

Concentration (μg/mL)	Hemolysis %
50	0
80	0
100	2
200	9

**Ciprofloxacin silica nanoparticles**	

**Concentration (**μ**g/mL)**	**Hemolysis %**

50	0
80	0
100	1
200	12

### Cytotoxicity assay

**Figure-4:**
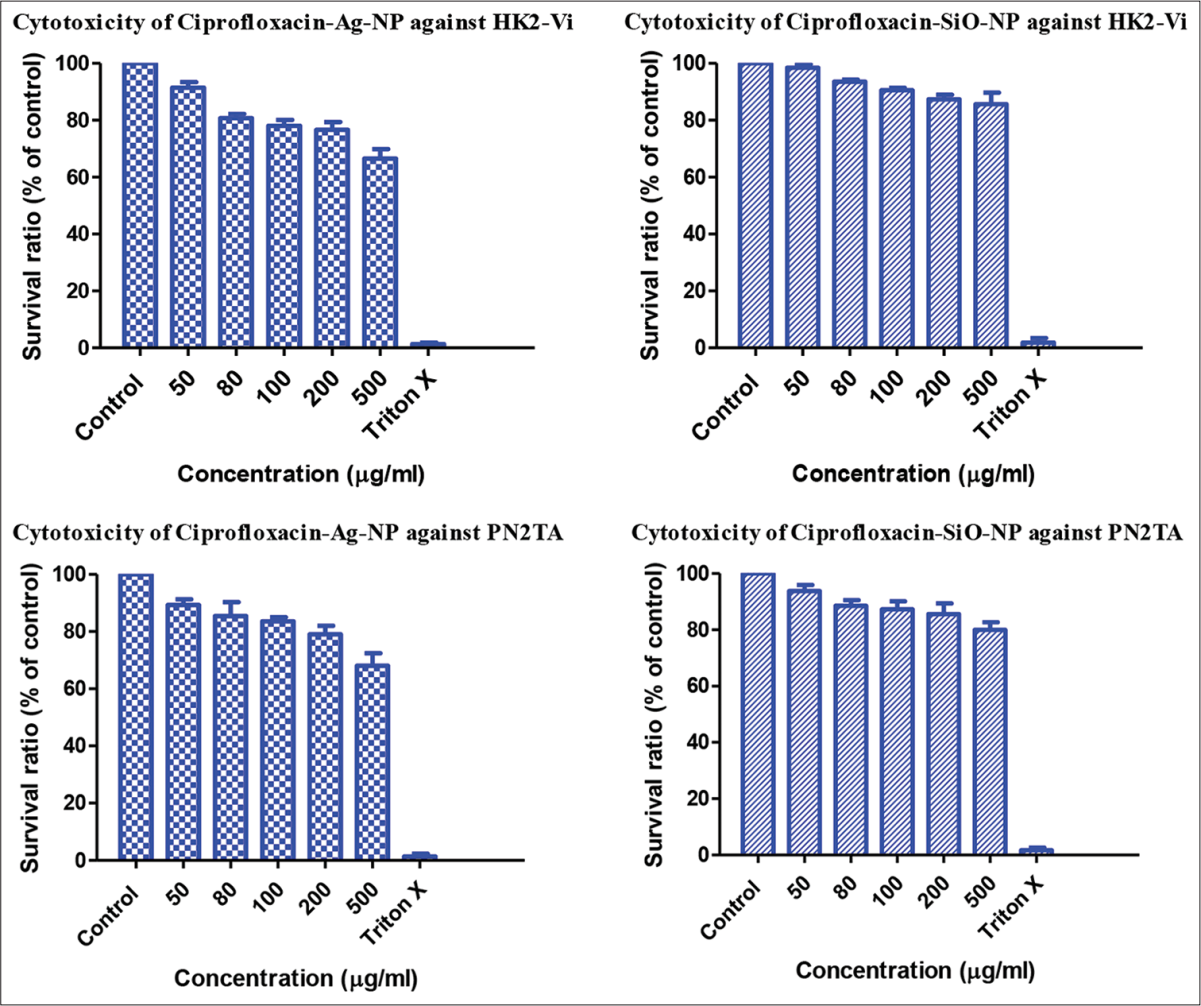
Cytotoxicity assay results for ciprofloxacin loaded to silica and silver nanoparticles.

As shown in Figure-4, ciprofloxacin loaded onto SiO-NPs had lower cytotoxicity on both pancreatic and kidney cell lines. When SiO-NPs were compared with the control in the presence of the HK2-Vi cell line at doses equal to or lower than 100 μg/mL, the percentage of cell viability was higher than 90%, whereas a slight reduction of cell viability was found at higher concentrations up to 500 μg/mL. On the other hand, after applying AgNPs to HK2-Vi cells, only a concentration equal to or lower than 50 μg/mL was able to maintain a cell viability percentage of more than 90%, whereas the remaining concentrations showed a massive reduction in the percentage of live cells of approximately 60% at a concentration of 500 μg/mL. Similarly, SiO-NPs were safer to use on the PN2TA cell line than AgNPs.

## Discussion

Antibiotic resistance resulting from pathogenic bacteria is the target of many research facilities and pharmaceutical companies, and nanotechnologies have proven to be a promising drug delivery method that can reduce the time required for the discovery of a new antibiotic and enhance the biological activity of the available one. After characterization of AgNPs and SiO-NPs, SiO-NPs were found to have a lower size with a mean diameter of 79.3 nm compared with AgNPs with a mean diameter of 180.2 nm. Many researchers have indicated that nanoparticles with a smaller size for the administration of antimicrobial agents enhance the penetration of microbial cells, leading to enhanced antimicrobial activity, as demonstrated in Table-3. Similarly, the PDI and zeta potential of SiO-NP were 0.43 and 30.7 mV, respectively, compared to 0.62 and 28.3 mV for Ag-NP, respectively. A PDI value in the range of 0.2–0.6 and a zeta potential value higher than 30 indicates a higher loading efficiency [21]. According to the MIC results presented in Table-2, both silver and SiO-NPs loaded with ciprofloxacin showed stronger biological activity than ciprofloxacin alone. The biological activity results were similar to those reported in a previously published paper in which nanoparticles improved the biological activity against *B. melitensis* Rev 1 [15]. Improvements in the biological activity of silver and SiO-NPs could be attributed to potential enhancements in both the pharmacokinetics and pharmacodynamics of loaded drugs [22, 23]. In addition, AgNPs have antibacterial activity through different mechanisms such as interaction with DNA and amino acids. In addition, it can reduce enzymatic activity in many cells by interaction with phosphorus and sulfur [13, 24, 25]. On the other hand, SiO-NPs are associated with the formation of reactive oxygen species, which have toxic effects on oxidative stress in different microorganisms [26]. The stronger antibacterial activity of ciprofloxacin loaded onto SiO-NPs could be attributed to the size differences compared with those of AgNPs. This finding is similar to previous literature findings showing an enhancement in biological activity in the presence of smaller nanoparticles [27–29]. The hemolytic activity of both nanoparticles was similar with a very low hemolytic activity, indicating a higher safety when applied to patients. Previous research has indicated that red blood cells are very sensitive to oxidative activity [30]. When comparing the safety of ciprofloxacin loaded to silver and SiO-NPs, SiO-NPs have been found to be safer than AgNPs even at a higher dose of 500 μg/mL silver. AgNPs have previously been found to exert cytotoxicity in different human cell lines [31]. SiO-NPs have also been used as a replacement for toxic nanoparticles because of their safety against human cells [32].

## Conclusion

Metallic nanoparticles are nanoparticle systems used by pharmaceutical companies and research laboratories to enhance drug delivery or increase drug potency. AgNPs were previously used for the delivery of ciprofloxacin against *B. melitensis* Rev 1. SiO-NPs can be used to replace AgNPs in the formulation of ciprofloxacin against *B. melitensis* Rev 1 according to the characterization of the nanoparticles, biological activity, and cytotoxicity. SiO-NPs loaded with ciprofloxacin had a smaller size, indicating better drug penetration to microbes, better PDI, and better zeta potential values, indicating better drug loading. In addition, SiO-NPs exhibit better biological activity and lower cytotoxicity.

## Authors’ Contributions

SAJ: Designed and performed the study, interpreted the results, and drafted the manuscript. MJS: Designed and performed the study and revised the manuscript. Both authors have read, reviewed, and approved the final manuscript.
